# Oral microbiota dysbiosis accelerates the development and onset of mucositis and oral ulcers

**DOI:** 10.3389/fmicb.2023.1061032

**Published:** 2023-02-09

**Authors:** Ziyang Min, Lei Yang, Yu Hu, Ruijie Huang

**Affiliations:** ^1^State Key Laboratory of Oral Diseases, Department of Pediatric Dentistry, National Clinical Research Center for Oral Diseases, West China Hospital of Stomatology, Sichuan University, Chengdu, China; ^2^Arts College, Sichuan University, Chengdu, China

**Keywords:** oral microbiota, dysbiosis, oral mucositis, recurrent aphthous stomatitis (RAS), probiotics

## Abstract

With the rapid development of metagenomic high-throughput sequencing technology, more and more oral mucosal diseases have been proven to be associated with oral microbiota shifts or dysbiosis. The commensal oral microbiota can greatly influence the colonization and resistance of pathogenic microorganisms and induce primary immunity. Once dysbiosis occurs, it can lead to damage to oral mucosal epithelial defense, thus accelerating the pathological process. As common oral mucosal diseases, oral mucositis and ulcers seriously affect patients’ prognosis and quality of life. However, from the microbiota perspective, the etiologies, specific alterations of oral flora, pathogenic changes, and therapy for microbiota are still lacking in a comprehensive overview. This review makes a retrospective summary of the above problems, dialectically based on oral microecology, to provide a new perspective on oral mucosal lesions management and aims at improving patients’ quality of life.

## Highlights

- We have produced an illustration representing the microbiota and its association with the oral cavity.
- The lotus leaf with lesions represents the subtle lesions and potential harm caused by dysbiosis, with the lotus flower expressing the health of oral mucosa.

## 1. Introduction

The human body is similar to the planet, composed of many eco-systems made up of micro-eco-systems. Therefore, it is not hard to understand that the oral cavity (an integral part of the human body) hosts a great variety of microorganisms. Each microbiota has its own microenvironments, and the resident microorganisms living in this microenvironment have a harmonious balance (homeostasis) of nutrients, byproducts, pH, and temperature distributed either synergetically or symbiotically (Bugshan et al., 2017). Alteration by local or systematic factors will cause imbalance or what we call “dysbiosis,” leading to the proliferation of potentially pathogenic microorganisms, e.g., prolonged inflammation (Bugshan et al., 2017).

The oral cavity is the main point of entry into the human body. It is constantly exposed to extrinsic and intrinsic stimuli, thus resulting in it acting as both physical and immunological barriers (Wang et al., 2019). The physical nature of the mucosal layer is physically preventing the intrusion of external stimuli and the immunological barrier of it, are the ability it detecting microbial antigens and triggering immune responses (Presland and Jurevic, 2002; Novak et al., 2008). The mucosal layer is multilayered epithelial cells with cell-cell junctions, and the oral cavity is a unique site where both soft and hard tissue exist. Its polymorphous surface harbors several highly heterogeneous microbial niches. Shifts in microflora will result in a domino effect in those microbial niches. Therefore, it could be speculated that mucosal signs and conditions can be transient, persistent, and intermittent under biological, physical, and chemical exposures. Failure to respond to or eliminate the stimuli will result in prolonged inflammation and more complicated situations. Take candidiasis as an example of ulceration. *Candida albicans* infection was associated with microbiota dysbiosis which will result in epithelial junction protein E-cadherin degradation and *C. albicans* invasion (Bertolini et al., 2019). Several studies (Bugshan et al., 2017; France and Villa, 2020; Gasmi Benahmed et al., 2021) have proposed various hypotheses on microorganisms’ involvement in the processes of oral lesions, hoping to provide clues for the target for the relief of oral mucosal diseases. This current review focuses on how oral microbiota dysbiosis is associated with oral ulcers and what are the molecular biologic mechanisms of this association. The goal of this review is to try to provide possible ideas for future oral ulcer research and treatment intervention.

## 2. Oral mucosal microbiota

### 2.1. Compositions of the normal oral mucosal microbiota

It has been proven that the occurrence of oral ulcers is closely related to the changes in the oral microbiota, such as shifts in the abundance of specific microorganisms and the emergence of new pathogenic microorganisms. Concurrently, the microbiota compositions in different oral habitats, such as mucosa, saliva, teeth, and tongue, are also different, which suggests that the microbiota changes in other oral habitats can influence the occurrence of ulcers, because saliva and chewing allow the microbiota of these habitats to interact with each other (Gao et al., 2018).

**FIGURE 1:**
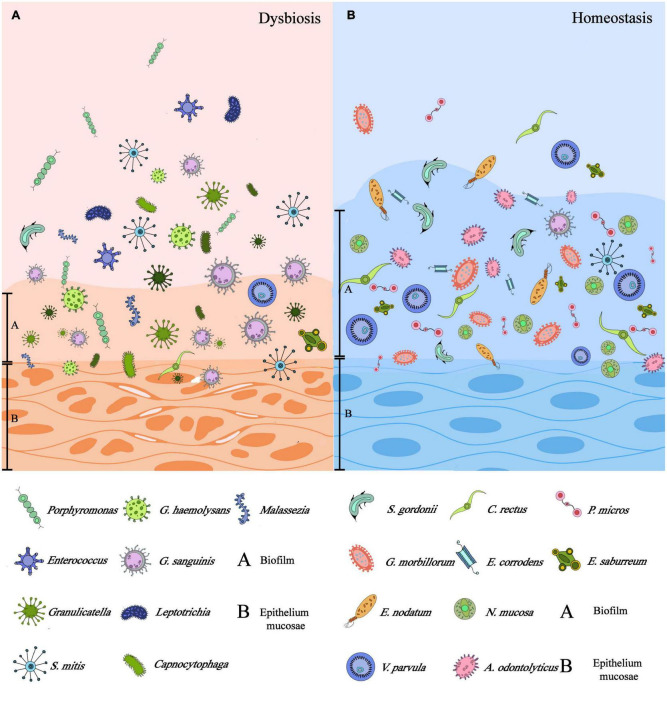
Differences between dysbiosis and homeostasis of mucosal microbiota: **(A)** the dysbiosis of microbiota could be symbolized by the impaired epithelium, thinner biofilm attached to the epithelium, and dominant microflora being pathogens. **(B)** Microbiota homeostasis could be characterized by intact epithelium, thicker biofilm, and commensal microflora instead. (This figure was partially cited from Lin et al. (2021) with modifications).

With the development of next-generation sequencing (NGS) and bioinformatics (Adami et al., 2021), more than 700 kinds of bacteria have been identified with various fungi, viruses, and protozoa in the oral cavity (Palmer, 2014). The Checkerboard DNA-DNA hybridization technique of the healthy host oral mucosal samples (comprised vestibule lip, attached gingiva, floor of mouth, buccal, tongue ventral, and hard palate) elucidated the abundance of native bacteria colonizing on these soft tissue surfaces, including *S. mitis, C. gingivalis, G. morbillorum, V. parvula, E. corrodens*, and etc. (Figure 1) (Mager et al., 2003). According to the Gram staining and bacterial morphology, the species of normal oral bacteria can be classified as follows:

Bac: Gram-positive:

1. Cocci–*S. mitis, S. oralis, S. constellatus, P. micros, S. sanguis, S. anginosus, S. intermedius*.
2. Rods–*G. morbillorum, A. odontolyticus, E. saburreum, A. naeslundii 1, P. acnes, A. israelii, E. nodatum, A. naeslundii 2, A. gerencseriae, S. gordonii*,

Bac: Gram-negative:

1. Cocci–*V. parvula*, *N. mucosa*.
2. Rods–*C. gingivalis, E. corrodens, P. melaninogenica, F. peridonticum, L. buccalis, F. nucleatum ss polymorphum, P. nigrescens, F. nucleatum ss nucleatum, P. intermedia, C. gracilis, C. showae, C. rectus, A. actinomycetemcomitans, T. denticola, P. gingivalis, B. forsythus, S. C. sputigena, C. ochracea*.

Fungi: *Aureobasidium_sp*, *Filobasidium_wieringae*, *Podoscypha_involuta*, *Candida_albicans*, *Malassezia_sp*, *Cladosporium_sp*, *Malas-sezia_globosa*, *Debaryomyces_hansenii, Nigrospora_sp, Tremello-mycetes_sp, Alternaria_armoraciae, Aspergillus_sp, Daedaleopsis_confragosa* (Stehlikova et al., 2019).

Protozoa: *Entamoeba gingivalis*, *Trichomonas tenax* (Zhou and Li, 2015; Deo and Deshmukh, 2019).

### 2.2. Oral mucosal microbiota dysbiosis and diseases

The microbiota imbalance and malfunction in the oral cavity is known as microbial dysbiosis. Studies have also suggested that specific microorganisms in the context of dysbiosis generate pathways that may potentially encourage the development of oral mucositis and prolonged the rehabilitation of existing ulcerations (Vasconcelos et al., 2016).

Clinical studies have used chemotherapy-induced mucositis patients as models for isolating bacterial strands, founding that the predominant strands were *G. haemolysans* and *Streptococcus mitis*, furthermore, the site where most bacterial changes were on the buccal mucosal layers (Napeñas et al., 2010). In lesions such as RAS (commonly found on the buccal, labial mucosa, and tongue areas), another study indicated that *Rothia dentocariosa* and *Acinetobacter johnsonii* are most likely to be linked with RAS and that the site of the most pronounced changes was the lower labial mucosa. Class *Clostridia* and genera *Lachnoanaerobaculum, Cardiobacterium* along with opportunistic pathogens such as *Leptotrichia*, and *Fusobacterium* increased in ulcerative samples, but a decrease in *Streptococcus salivarius*, *Neisseria*, and *Veillonella* species (Stehlikova et al., 2019). In active ulcers, the fungi *Malassezia* has its dominance followed by *C. albicans*, and the association between *Malassezia* and *S. salivarius* and *Haemophilus* is negative, whereas, the association is believed to be positive in *Porphyromonas*. The association between *A. johnsonii* and active ulceration was positive in the study, besides, an increase in *A. johnsonii* in the ulcerative site was also observed with a greater amount of *Haemophilus parahaemolyticus* or *H. parainfluenza* (Stehlikova et al., 2019).

In their experiments, Wang et al. (2019) concluded that the α diversity in normal and RAS patients was similar, but the fundamental microbial compositions of the two showed significant differences, embodying as that *Firmicutes* on the phylum level, *Bacilli* on the class level, *Lactobacillales* on the order level, *Streptococcaceae* on the family level, and *Streptococcus* on the genus level were prominent in the normal, whose result echoed the above findings that there was more taxonomic abundance of *Streptococcus* in normal patients than in RAS. In contrast, RAS patients exert more abundant *Enterobacteriales* on the order level, *Enterobacteriaceae* on the family level, *Escherichia* and *Alloprevotella* on the genus level, and *E. coli* on the species level. In summary, increased *E. coli* and *Alloprevotella* and decreased *Streptococcus* in microbial communities will most likely be associated with RAS.

### 2.3. Defensive roles of normal oral microbiota

The presence of oral mucosal commensal microbiota is important to the establishment of protective/homeostatic immune responses as it provides low dosage of pathogenic stimulation. This stimulation could sustain the activity of normal functional immune system.

The lipopolysaccharide (LPS) of Gram-negative bacteria can effectively activate toll like receptor 4 (TLR4) through the pathway of vesicular acidification. Meanwhile, the activation is weakened during the second induction to maintain the microbial immune homeostasis of the oral mucosa (Wu et al., 2015; Kantrong et al., 2019). Similarly, TLRs also mediate immune regulation toward fungi and viruses (Groeger and Meyle, 2019). Interleukin-17 (IL-17)-expressing cells (type 17 cells) also play an essential role in pathogen-associated molecular patterns (PAMPs) and inflammation recognition and response in oral mucosa through the binding of expressed C-C chemokine receptor 6 (CCR6) to mucosal C-C chemokine ligand 20 (CCL20) and the binding of IL-17 to its heterotrophic receptor (IL-17RA and IL-17RC) found mainly in epithelial cells (Acosta-Rodriguez et al., 2007; Li et al., 2019).

From the perspective of metabolites, nitric oxide (NO) produced as the metabolic products of oral microbiota exerts a positive influence on the prevention and control of oral diseases as it could activate macrophages, and then proceed with macrophage inhibition and phagocytosis of bacteria (Brennan et al., 2003; Hezel and Weitzberg, 2015; Bandara et al., 2019). Meanwhile, bacteriocin and hydrogen peroxide produced by some bacteria can antagonize other bacteria (Heilbronner et al., 2021). Rosier et al. (Maekawa et al., 2014) have confirmed that *Porphyromonas gingivalis* can affect the clearance of bacteria and inhibit the immune response. There are also inverse relationships in fungi, for instance, *Pichia* can inhibit the growth of potential pathogens, such as *Candida*, *Aspergillus*, and *Fusarium* (Baumgardner, 2019).

At the population level, the oral microbiota with higher α diversity can retain a higher capacity of resistance to invasive microorganisms and antibiotics. In contrast, extremely low β diversity of oral microbiota indicates that the microbiota of different hosts is highly homogenous and conserved (D’Agostino et al., 2022; Xu et al., 2022). The α diversity and salivary compositions assure that despite other influential factors, it will have certain degrees of buffer and resistance to assemble residency microbiota’s core functions in hosts without much variation in its microbial compositions (Rosier et al., 2018).

## 3. Influencing factors of oral mucosal microbiota shifts

### 3.1. Smoking

**FIGURE 2:**
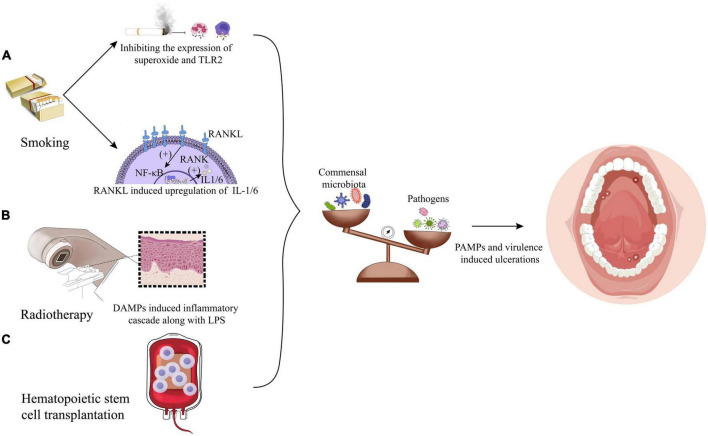
Factors leading to the microbiota shifts: **(A)** smoking exerts a significant influence on dysbiosis by microbiota carried by immune reaction inhibition and upregulation of IL-1/6 induced by receptor activator of nuclear factor- κβ Ligand (RANKL). **(B)** Radiotherapy could initiate oral mucositis induced by damage-associated molecular patterns (DAMPs) along with lipopolysaccharide (LPS). **(C)** Hematopoietic stem cell transplantation impacts the flora alterations in a way undiscovered.

It has been reported that the number of smokers around the world is 1.14 billion, and the number of males is about five times that of females. Besides, about 30% of global smokers were from China in 2019 (Zhou et al., 2019; GBD 2019 Tobacco Collaborators, 2021). Smoking can inhibit the phagocytosis of macrophages and neutrophils by inhibiting bacterial stimulated expression of superoxide and surface receptors (e.g., TLR2), thus weakening the clearance of newly colonized microorganisms. Meanwhile, smoking may also induce the receptor activator of nuclear factor- κβ Ligand (RANKL) to upregulate the levels of IL-1 and IL-6, affecting the oral immune response to the microbiota (Matthews et al., 2012; Ahmed et al., 2021), as shown in Figure 2. In addition, smoking also changes the microbiota in the oral mucosa by affecting biofilm formation and adhesions of specific microorganisms (e.g., *Staphylococcus aureus* and *Streptococcus pneumoniae*) (Kulkarni et al., 2012; Mutepe et al., 2013). Studies have found that compared with non-smokers, smokers have higher oral microbiota α diversity with *Firmicutes*, *Bacteroidetes* and *Actinobacteria* significantly increasing, but the relative abundance of phyla *Bacteroidetes*, *Fusobacteria*, *Proteobacteria*, *Neisseria*, *Branhamella*, *Porphyromonas*, and *Gemella* was decreased (Morris et al., 2013; Vallès et al., 2018; Huang and Shi, 2019). It has been reported that compared with non-smokers, smokers are more prone to actinomycosis and have more significant levels of fugal CFU/ml (Abduljabbar et al., 2017; Angst et al., 2019). However, smoking is not the fundamental cause of RAS. Sawair (2010) found that smoking might increase the thickness of the squamous epithelial cell layer to reduce ulcer occurrence. In short, smoking does not show a causal relationship with the occurrence of ulcers, but the impact of smoking on oral mucosal microbiota and immunity does have a repercussion on the progress of ulcers and mucositis from other aspects.

### 3.2. Radiotherapy

Radiotherapy and chemotherapy are the most commonly used methods for treating head and neck tumors in addition to surgery. However, more than 80% of patients will develop oral mucositis after radiotherapy, namely, radiation-induced oral mucositis (RIOM) (Al-Dasooqi et al., 2013; Bockel et al., 2018). Therefore, it is of clinical value to explore whether radiotherapy accelerates the occurrence of oral ulcerations and mucositis from the perspective of microbiota dysbiosis to improve patients’ prognosis and quality of life. The research of Hou et al. (2018) shows that radiotherapy has no significant effect on the overall microbiota abundance, but it has a dose-dependent effect on the specific components of the microbiota. At the level of phylum, with the increase of radiation dose, the relative abundance of *Proteobacteria* and *Spirochaetes* increases significantly, while *Fusobacteria* decreases. At the genus level, the relative abundance of *Prevotella*, *Fusobacterium*, *Leptotrichia*, *Campylobacter*, *Peptostreptococcus*, and *Atopobium* decreases, while *Pseudomonas*, *Treponema*, *Granulicatella*, and *Capnocytophaga* shows an upward trend (Hou et al., 2018). Another study on patients of oral cancer after radiotherapy showed that the increase of *S. aureus* and *P. aeruginosa* in oral mucosa after radiotherapy was statistically significant, while *Escherichia coli* dropped to a lower level after 6 weeks of radiotherapy (Subramaniam and Muthukrishnan, 2019). Stringer et al. further found that damage-associated molecular patterns (DAMPs) induced by radiotherapy, such as HMGB1, can bind to and activate TLR4. This leads to the initiation of the inflammatory cascade through interaction with bacterial LPS, which is shown in Figure 2. TLR4 signaling pathway can stimulate host cells to produce proinflammatory cytokines, such as tumor necrosis factor-α (TNF-α), thereby forming a full-thickness ulcer in the oral mucosa epithelium cell layers (Stringer and Logan, 2015). However, in combination with the role that TLR4 could induce basic protective immunoreaction, TLR4 plays dual roles in protection and injury; thus, it might be a drug-targeting site to prevent dysbiosis-induced ulcer deterioration.

### 3.3. Hematopoietic stem cell transplantation

Hematopoietic stem cell transplantation (HSCT) is a common treatment for hematological diseases. Almost 60–80% of the patients who receive treatment will develop oral mucositis (Villa and Sonis, 2016). It is worth noting that more and more studies have shown that HSCT can lead to oral microbiota dysbiosis and exaggerate inflammatory status (Hou et al., 2018; Laheij et al., 2019; Miranda-Silva et al., 2020), therefore, it is hypothesized that relieving microbiota dysbiosis would contribute to reduce the HSCT treatment complications. Previous studies have shown that bacteria and fungi, such as *P. gingivalis*, *Enterococcus* sp., and *Candida* are all associated with the occurrence of RAS after HSCT treatment (de Mendonça et al., 2012; Osakabe et al., 2017). Besides, Laheij et al. (2019) found that 3 months after the termination of HSCT treatment, the oral microbiota of the patients with RAS almost recovered to the same level as before. But within one or 2 weeks after the termination of HSCT treatment, there were significant component changes. Overall, *Streptococcus australis*/*parasanguinis*/*OT057*/*OT066*, *Veillonella atypica*/*dispar*, genus *Actinomyces*, *Actinomyces sp*. *OT172*, *Actinomyces graevenitzii*, and *Gemella haemolysans*/*morbillorum*/*sanguinis* are predominant before treatments, while *S. aureus*/*caprae*/*epidermidis/warneri*, *Scardovia wiggsiae*, *Enterococcus faecalis*, and *Lactobacillus fermentum* superseded the main species of the changed flora under HSCT treatment, although there is no significant changes in fungi (Laheij et al., 2019). Furthermore, the study by Muro et al. (2018) elucidated that after HSCT treatment, *Staphylococcus spp., Enterococcus spp.*, and *Lautropia mirabilis* of, which are rarely detected in normal mucosal microbiota, were identified. The research of Ohbayashi et al. (2021) also showed that the normal flora of oral mucosa decreased to 25.5% after HSCT treatments, and the flora is mainly composed of coagulase-negative *Staphylococci* (39.4%), *Enterococcus* sp. (11.7%), *Candida* sp. (9.6%), and *Stenotrophomonas maltophilia* (2.1%), which was different from that before. However, the limitation of the above research is that chemotherapy or antibiotics will inevitably be used in HSCT treatments, and it is still challenging to determine whether HSCT is a direct factor in the alteration of the oral flora or not. Therefore, only when mouse models received only HSCT was created, the single factor affected microbiota variables can be obtained in turn.

Ultimately, it is unclear whether changes in oral microbiota caused by smoking, radiation, and HSCT are the causes or influencing factors of oral ulcers, with the timeline and correlation between the change of colony and the occurrence of ulcers to be evaluated. Nevertheless, the impact of the change of colony on the development and healing process of ulcers is undeniable; thus, the above research results are still of great significance in guiding ulcer treatments.

## 4. Immunol changes caused by mucosal microbiota dysbiosis

### 4.1. Pathologic staging of mucositis development

Research conducted by Hong et al. (2019) explained that patients under chemotherapy would have dysbiosis-mediated mucositis due to increasing inflammatory factors released into the oral cavity. Moreover, tissue damage will cause the release of reactive oxygen species (ROS) and their DNA materials. Then DNA breakage will lead to the activation of apoptotic pathways, which are regulated by p53 activation, caspase-3 elevation, and endogenous DAMPs (Triarico et al., 2022). The process mentioned above is the so-called phase I of mucositis development, synchronously marking the initiation of phase II mucositis. During phase II, inured mucosal cells promoted transcriptional factors such as nuclear NF-κB, which is an essential transcriptional mediator that governs over 200 genes associated with various proinflammatory cytokines involved in the early degradation of connective tissue, endothelium, and adhesion molecules apoptosis (e.g., TNF-α, IL-6, and IL-1β) (Triarico et al., 2022).

**FIGURE 3:**
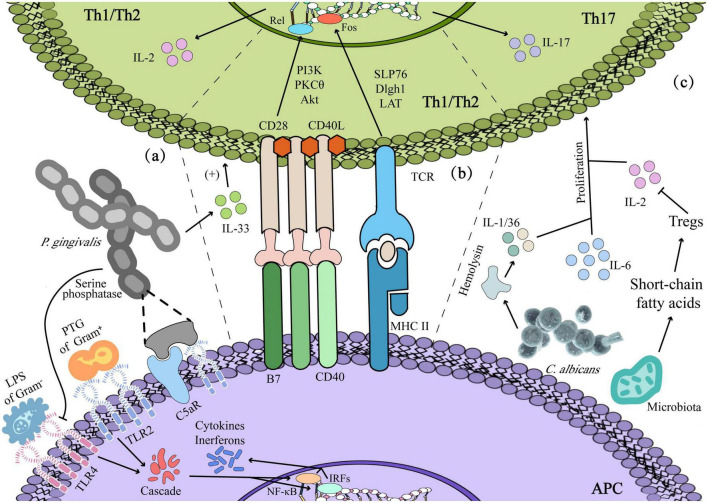
Dysbiosis-induced immunoreaction: **(a)** innate immunity: lipopolysaccharide (LPS) and Peptidoglycan (PTG) contribute to the activation of immune and inflammatory responses in Th1/2. **(b)** Adaptive immunity: *Porphyromonas gingivalis* enhanced Th2 cytokine-mediated inflammatory immune response by prompting the secretion of IL-33 of epithelium, and antigen presentation by antigen–presenting cells (APCs) could activate Th1/2 by signal 1 and signal 2. **(c)** *Candida albicans* producing cytokines such as IL-1/IL-36 mediates the expression of IL-17 and the proliferation of Th17 cells, and short-chain fatty acids produced by microbiota facilitate the viability of Th17 with regulating the Tregs.

Meanwhile, other signaling pathways work coordinately by activating a positive feedback loop system, which is marked as phase III. TNF-α initiates mitogen-activated protein kinase (MAPK) activation on targeted cells while sustaining NF-κB processes. MAPK signaling pathways also mediate caspase-3 activation. Usually, at this stage, ulceration will be protracted as oral dysbiosis and leads to additional proinflammatory cytokines released by infiltrating mononuclear cells (phase IV). It is in phase IV that former studies have shown that cytotoxic T lymphocytes (CTLs) contributed to oral mucosal epithelial cells apoptosis, and enriched pathogens with their antigens at the ulcerative mucosa, such as LPS and hemolysin, can stimulate the host to produce a proliferative response (PR) of peripheral blood mononuclear cells (PBMCs) and T cells, then CTLs infiltrate oral mucosal epitheliums and lamina propria to deteriorate the mucosal damage (Sun et al., 2002; Suárez et al., 2020). In consequence, it can be seen that both innate immunity and adaptive immunity play important roles in dysbiosis-mediated oral ulcers. The lesions are believed to start repair during the phase V healing stage, which is characterized by epithelial cell proliferation and cellular differentiation aiming at restoring epithelial cells’ integrity (Singh and Singh, 2020). Subsequently, this section will elaborate on the role of oral dysbiosis in oral ulcers from the perspective of innate immunity and adaptive immunity shown in Figure 3.

### 4.2. Innate immunity response

Peptidoglycan (PTG) and LPS are the components of bacteria that can act as ligands for TLR2 and TLR4, respectively, to activate PAMPs and then the innate immune system. Meanwhile, macrophages also respond to antigen stimulation by upregulating the expression of membrane TLR2/4 (Liang et al., 2013; Dabbagh et al., 2014). In Behçet’s disease (BD) patients with oral ulcers, the oral microbiota seems to lead to the excessive persistence of inflammation by inducing the abnormal polarization of M1/M2, which is related to the reduction of CCR1 of M2 in BD patients, with CCR1 attenuating the chemotaxis effect of M2 on MIP-1α and decreasing the expression of IL-10. Eventually, the macrophages at the ulcerative site gradually change into the M1 dominant paradigm, that is, inflammatory injury type (Hirahara et al., 2022). Rosier et al. ulteriorly have confirmed that *P. gingivalis* can cause crosstalk between the C5a receptor (C5aR) and TLR2, thereby increasing the inflammatory response, affecting the clearance of other bacteria and interfering with the healing. In the meantime, *P. gingivalis* can also inhibit the immune response by secreting a kind of LPS and serine phosphatase to inhibit the expression of TLR4 and IL-8 (Maekawa et al., 2014). Moreover, a large number of *S. sanguinis*-like microorganisms in the ulcers of RAS patients can promote Langerhans cells to present the bacteria-induced homologous peptides within epithelial heat shock proteins and then act as a bridge to activate humoral and cellular immunity simultaneously (Stehlikova et al., 2019).

### 4.3. Adaptive immunity response

In terms of humoral immunity, Stehlikova et al. (2019) found that anti-*M. timidum* IgG and anti-*C. albicans* IgA was significantly elevated in patients with RAS, indicating that specific microorganisms at the ulcer activated humoral immunity, and IgG and IgA secreted by B2 cells further induced antibody-dependent cell-mediated cytotoxicity (ADCC) to clear pathogenic bacteria. In cellular immunity, Groeger and Meyle (2019) illustrated that *P. gingivalis* could mediate epithelial cells to enhance the secretion of IL-33, thereby enhancing Th2 cytokine-mediated inflammatory immune response. Otherwise, intercellular adhesion molecules (ICAM), such as carcinoembryonic Ag-related cell adhesion molecule 1 (CEACAM1), are highly expressed in the junctional epithelium. They serve as receptors for various bacterial antigens, for instance, CEACAM1 will further interact with infiltrating polymorphonuclear (PMN) and T cells, ultimately enhancing the cytotoxicity (e.g., Perforin) of T cells and NK cells (Suárez et al., 2021).

In a healthy state, mechanical injury such as chewing can make oral mucosa produce IL-6 and promote the basic proliferation of Th17 (Kimura and Kishimoto, 2010). However, the activation of Th17 in oral diseases is more dependent on the change in microbiota. For instance, excessive hemolysin produced by *C. albicans* induces cell damage and c-Fos-driven intrinsic inflammatory reaction, during which damaged cells produced cytokines such as IL-1/IL-36, sequentially mediating the expression of IL-17 and the proliferation of Th17 cells (Conti and Gaffen, 2010). Besides, oral microbiota regulates Tregs and Th17 cells by producing short-chain fatty acids, whereby Tregs enhance the activity of Th17 cells by consuming IL-2 and reverse the inhibitory effect of IL-2 on Th17 cell differentiation, thereby, enhancing the Th17-mediated clearance of *C. albicans* in the oral cavity (Gaffen and Moutsopoulos, 2020).

### 4.4. Oral mucosal barrier detriment

The current hypothesis suggests factors: genetic predisposition, viral, bacterial, microelement deficiencies, hormonal, stress, mechanical stimuli, food allergies and immuno-deficiencies to be strongly associated with oral mucosal diseases (OMD) (Alrashdan et al., 2016; Abduljabbar et al., 2017; Vila et al., 2020). These factors, however, are highly correlated with microbiota dysbiosis in the oral cavity. Genetic expression analysis for patients under chemotherapy has found that during chemotherapy-induced dysbiosis, the abundance of *F. nucleatum* is increased at the mucositis lesion sites. This increase of *F. nucleatum* will provoke significant upregulation of TNF, CCL20, IL-17C, CXCL2, PMAIP1, DEFB4A, and DEFB103A, among which TNF could activate NF-κB and trigger apoptosis or necroptosis of epithelium *via* its extrinsic pathways (Hong et al., 2019). Another category of genes that may have a role in the induction of mucosal cell apoptosis in this process was the up-regulation of PMAIP. According to Hong et al. (2019) it was the gene encoding protein NOXA [it is a protein of proapoptotic Bcl2 homology 3 (BH3)], then it triggers a series of cascade-leading cell deaths. As mentioned previously, candidiasis is a common lesion for patients with compromised immune systems. When there is a disruption in the microbial equilibrium, i.e., immunosuppression activities, certain bacteria (such as *Enterococcus* species) will overgrow and establish a mutualistic network with *C. albicans*, and this change or dysbiosis will prolong the inflammatory responses and amplifies mucosal damage (Bertolini et al., 2019).

## 5. The future of microbiota in mucositis relief: Bench to bedside

**FIGURE 4:**
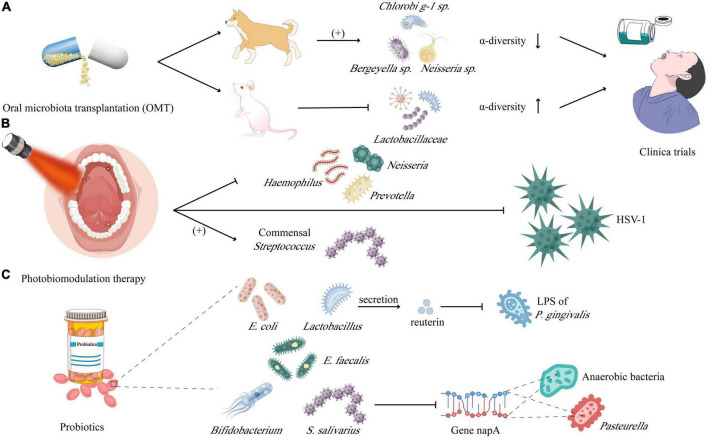
Microbiota in prevention and therapy of mucositis and ulcerations: **(A)** oral microbiota transplantation (OMT) was carried out in dogs and found that the relative abundance of *Chlorobi g-1 sp.*, *Neisseria sp.*, and *Bergeyella sp.* increased rather than α-diversity; while downregulating the *Lactobacillaceae* but increasing the α-diversity in mice. **(B)** Photobiomodulation therapy is demonstrated to inhibit the *Haemophilus*, *Neisseria*, and *Prevotella*, instead benefiting the commensal *Streptococcus*, meanwhile which is lethal to HSV-1, too. **(C)** Probiotics including *Lactobacillus, Bifidobacterium, Escherichia coli*, and *Escherichia faecalis*, could neutralize the lipopolysaccharide (LPS) of *Porphyromonas gingivalis* by secreting the reuterin and inhibiting *Pasteurella* and anaerobic bacteria by downregulating the expression of gene encoding nitrate reduction (napA).

Compared with oral microbiota, the research on gastrointestinal microbiota is more in-depth. A recent review based on regulating microbiota to treat diseases mentioned that the intake of fecal microbiota transplantation (FMT), symbolic microbiological consortia, prebiotics, microbiota-derived proteins, and metabolites is expected to be effective in reconstructing a healthy microbiota (Sorbara and Pamer, 2022). This section focuses on the emerging methods for treating oral ulcers through microbiota regulation from three aspects: oral microbiota transplantation (OMT), probiotics, and photodynamic therapy (Figure 4).

### 5.1. Oral microbiota transplantation (OMT)

Compared with FMT, which has been widely used in clinics, the research on OMT is still constrained to animal experiments. Beikler et al. (2021) performed OMT in dogs with periodontitis, there is almost no difference in the abundance of the microbiota between the treated and control groups before receiving OMT. While 2 weeks after receiving OMT, the α-diversity of the oral microbiota of the treated group was significantly lower than that of the control and was most similar to the oral microbiota of the donor, with the relative abundance of ASV40 (Chlorobi g-1 sp.), ASV150 (Neisseria sp.) and ASV44 (Bergeyella sp.) increased. Another study (Xiao et al., 2021) in mice with RIOM showed that the level of IL-1/6 and TNF-α in tongue tissue and plasma of mice decreased after OMT treatments. From the perspective of microbiota, OMT reversed the rise of *Lactobacillaceae* in RIOM and gave the oral microbiota higher α-diversity. Later research further showed that S100A9 decreased in RIOM, but significantly increased after OMT, which might indicate that the level of S100A9 plays an important indicative role in both inflammation level and cancer prognosis (Xiao et al., 2021). The clinical application of OMT depends on a variety of factors, including the immune function of the recipient, the composition of the oral microbiota of the recipient, the screening of the healthy microbiota of the donor, and the pathogen screening of the donor microbiota (Beikler et al., 2021; Xiao et al., 2021). Accidentally, transplantation of pathogenic microorganisms could cause serious consequences such as periodontitis, caries, or even inflammatory bowel disease (IBD), arthritis, colorectal and pancreatic cancers, and Alzheimer’s disease (Tuganbaev et al., 2022), so OMT is not a long-term solution (Nascimento, 2017). Therefore, symbolic microbial consortia and engineered symbolic bacteria may become more targeted, universal, and ideal development directions.

### 5.2. Photobiomodulation therapy

Photobiomodulation therapy (PBMT) has gradually become a potential therapeutic method for many oral diseases, such as oral lichen planus (OLP), RAS, hyperpolarization, trigeminal neuralgia (TN), etc. PBMT can achieve anti-inflammatory and analgesic functions, promote wound healing and regeneration of damaged peripheral nerve tissues through the photoreceptors of mitochondria and Ca^2+^ channels of cell membranes (Kalhori et al., 2019), and there are no apparent side effects (Özberk et al., 2018). However, the specific effects of PBMT on oral microbiota need further elucidation. Some studies have shown that PBMT can form cytotoxic and highly lethal single oxygen to kill bacteria by laser excitation in the presence of endogenous oxygen (Kwiatkowski et al., 2018). A study on the application of PBMT on acute necrotizing ulcerative gingivitis treatment showed that the overall diversity of oral microbiota decreased after treatment, and the number of *T. microdentium, F. nucleatum*, and *P. intermedia* all decreased (Siddiqui et al., 2020). In addition, Zanotta et al. carried out a more in-depth test in rats, which showed that topical PBMT after indocyanine green (ICG) washing could produce a strong killing effect against Gram-positive bacteria and drug-resistant bacteria such as methicillin-resistant *S. aureus*, thereby inhibiting inflammation and promoting ulcer healing and fibrosis (Motamedifar et al., 2021). After PBMT for patients with RIOM, it was found that the number of pathogenic bacteria in the oral mucosa of patients decreased, including *Haemophilus* (from 15 to 11%), *Neisseria* (from 11 to 8%), and *Prevotella* (from 32 to 24%). However, it was accompanied by the increase of commensal bacteria *Streptococcus* (from 13 to 22%), which seems to indicate that the bactericidal effect of PBMT is selective and needs to be further explored (Zupin et al., 2018). In addition to bacteria, PBMT can also facilitate to repair oral ulcers caused by HSV-1 (Zupin et al., 2018). Owing to different light wavelengths having different antiviral mechanisms, the red laser may activate the antiviral immune response and inhibit virus replication, while the blue laser still needs to be further explored (Zanotta et al., 2020). In summary, compared with antibiotics, photosensitizers combined with PBMT may be very promising in microbial dysbiosis control, but the photosensitizer dose and light wavelength with unified standards still need to be clarified further. In addition, the oral mucosa of the recipient is required to have as low microbial abundance as possible before OMT, so PBMT combined with OMT may become a powerful method to treat oral ulcers in the future.

### 5.3. Probiotics

Probiotics can regulate the oral microbiota by colonizing the oral mucosa, producing specific metabolites to maintain the ecological balance of the host microbiota, and achieving a healthy state of moderate immunity (Sarao and Arora, 2017). This is different from the OMT mentioned above or symbolic microbiological consortia. For example, the introduction of probiotics is usually limited to a few species, without the disadvantage of introducing pathogenic bacteria. Simultaneously, taking probiotics is not to rebuild the flora, but to regulate the whole flora through the action of a few bacteria, therefore, the original microbiota of the subject is greatly preserved. Probiotics mainly include *Lactobacillus, Bifidobacterium, E. coli*, *E. faecalis*, and etc. It has been proven that probiotics can inhibit the growth, adhesion, proliferation, and biofilm formation of *C. albicans* (Janczarek et al., 2016; Hoare et al., 2017). Furthermore, Han et al. found that compared with pathogenic bacteria alone or the control group, the palate lesion treated with the mixture of *L. reuteri* (probiotic) and *P. gingivalis* (pathogen) has a faster healing rate. This phenomenon can be explained as reuterin produced by *L.* reuteri could degrade LPS of *P. gingivalis*, which ulteriorly inhibits LPS induced NLRP3 inflammasome (Han et al., 2020). Last but not least, Wang et al. (2021) tested in the mice of RIOM and found that *S. salivarius* K12 partially reconstituted the oral mucosa microbiota of RIOM mice, resulting in a significant reduction of *Pasteurella* and anaerobic bacteria by downregulating the expression of the gene encoding nitrate reduction (napA).

## 6. Conclusion

Oral microbiota dysbiosis is a multi-factor and multi-dimensional shift of the native microorganisms in the oral cavity. This microbiota shift will replace mutualistic/symbiotic microorganisms with potentially pathogenic microorganisms. The exact bacterial strands change in the microflora during the different onset and development stages of oral lesions is unknown, leaving room for future quantitative populational studies to be carried out. From the perspective of etiologies, medical treatments such as chemotherapy, antibiotics, and prosthodontic repairments can potentially also induce dysbiosis; thus, knowing the necessity of different risk factors will aid patient prognostic management (Brantes et al., 2019; Hong et al., 2019; Aguglia et al., 2020; Cardona et al., 2020; Neelakantan and Solomon, 2022). Based on current studies, once microbiota shifts participate in the progress of oral lesions, this dysbiosis will provide the foundation for the subsequent viral and fungal infections, such as *Histoplasma capsulatum*, cytomegalovirus, herpes simplex virus, *Trichomonas spp*, *Leptospira, Treponema pallidum*, etc. (Figueira et al., 2017; Folk and Nelson, 2017; Ito et al., 2019; Chen and Wang, 2020; Dmytrenko et al., 2020; Keah et al., 2022), which is principally attributed to their independent virulent factors, thereby they are not ulteriorly clarified in this review.

Microbiota dysbiosis may also lead to epigenetic changes by modification of genes and histones, regulation of non-coding RNA, etc., with gene serotonin transporter gene (SLC6A4), mannose-binding lectin 2 (MBL2), and so forth being susceptible to alterations of oral flora, whose molecular mechanism needs to be further ascertained (Bankvall et al., 2020; Slezakova et al., 2020; Baioumy et al., 2021; Bartakova et al., 2022). In the aspects of remedy of oral flora, some experiments proposed new approaches and hypotheses for utilizing herbal medicines or nutrients like curcumin, garlic, aloe vera, and oregano oil, which might be a promising domain of research in the future (Mahattanadul et al., 2018; Hoglund et al., 2020; Shi et al., 2020; Hosny et al., 2021). Our review focused mainly on oral mucositis RAS; however, mechanical trauma and psychological factors induced oral lesions are not mentioned; whether these proposed factors will influence microbiota shifts also remains to be explored.

## Author contributions

ZM and LY contributed to the conception and design of the work, drafting the manuscript, made final approval of the version to be published, and agreed to be accountable for all aspects of the work in ensuring that questions related to the accuracy or integrity of any part of the work are appropriately investigated and resolved. YH contributed to the interpretation of data for the work, made the figures, drafting the manuscript, made final approval of the version to be published, and agreed to be accountable for all aspects of the work in ensuring that questions related to the accuracy or integrity of any part of the work are appropriately investigated and resolved. RH contributed to the conception and design of the work, revised the manuscript, made final approval of the version to be published, and agreed to be accountable for all aspects of the work in ensuring that questions related to the accuracy or integrity of any part of the work are appropriately investigated and resolved. All authors contributed to the article and approved the submitted version.
